# The Influence of Simulated Organic Matter on the Inactivation of Viruses: A Review

**DOI:** 10.3390/v16071026

**Published:** 2024-06-26

**Authors:** Christina Allingham, Miyu Taniguchi, Amanda J. Kinchla, Matthew D. Moore

**Affiliations:** 1 Department of Food Science, University of Massachusetts, Amherst, MA 01003, USA; miyu.taniguchi@marusanai.co.jp (M.T.); amanda.kinchla@foodsci.umass.edu (A.J.K.); 2 Department of Food Science and Technology, Tokyo University of Marine Science and Technology, Minato City, Tokyo 108-8477, Japan

**Keywords:** foodborne viruses, organic load, inactivation, norovirus, disinfection

## Abstract

Viruses impose a significant public health burden globally, and one of the key elements in controlling their transmission is the ability to inactivate them using disinfectants. However, numerous challenges to inactivating foodborne viruses exist due to inherent viral characteristics (such as recalcitrance to commonly used inactivation agents) and external factors (such as improper cleaning before application of inactivation agent, improper contact time, etc.). Given the potential for improper application of disinfectants (such as shorter than recommended contact time, improper disinfectant concentration, etc.), understanding the performance of a disinfectant in the presence of an organic load is important. To accomplish this, the introduction of simulated organic loads is often used when studying the efficacy of a disinfectant against different viruses. However, the different types of simulated organic loads used in foodborne virus inactivation studies or their relative effects on inactivation have not been reviewed. The purpose of this review is to survey different simulated organic load formulations used in studying foodborne virus inactivation, as well as present and compare the influence of these different formulations on viral inactivation. The findings included in this review suggest that many simulated organic load formulations can reduce disinfectants’ efficacy against viruses. Based on the findings in this review, blood, particularly serum or feces, are among the most commonly used and efficacious forms of simulated organic load in many tests.

## 1. Introduction

Viruses continue to pose a significant public health burden and have been estimated to comprise a large portion of human infections [1]. Acute gastroenteritis is one of the most frequent health issues in the world, especially in children; it causes acute symptoms of abdominal pain, cramping, nausea, vomiting, and diarrhea [2,3]. Although vaccines and antiviral medications are commonly used for controlling viral diseases such as hepatitis A virus (HAV; a member of the *Picornaviridae* family), and rotavirus (a member of the *Reoviridae* family) many do not exist for many common enteric viruses [4]. Norovirus, a member of the *Caliciviridae* family, is one of the most frequent causes of foodborne illness globally, placing a considerable burden on health systems and food industries [5]. One of the challenges in controlling foodborne virus transmission is their ability to persist in foods and the environment, resulting in a notable amount of environmental transmission in addition to transmission via foods and direct person-to-person contact [6,7]. It is important to note that the behavior of non-enveloped viruses (such as norovirus) versus an enveloped virus (such as human immunodeficiency virus HIV and respiratory syncytial virus (RSV) differs. Non-enveloped viruses lack a membrane envelope, which traditionally results in a higher stability against disinfection methods [8]. Therefore, proper disinfection (such as using recommended contact time, concentration of the disinfectant, and good manufacturing practices) and implementation of effective sanitation protocols are important in reducing virus transmission, especially in the case of non-enveloped viruses. An extensive list of commonly used disinfectants and their respective inactivation abilities on viruses can be found in Table 1.

Chemical disinfectants such as sodium hypochlorite and quaternary ammonium compounds are most used and relied upon in hospitals, laboratories, food preparation facilities, and many other industrial, institutional, or domestic settings in the control of foodborne virus transmission [13,14]. However, many commonly utilized disinfectants lack ideal efficacy (4–5 log reduction) against viruses, and disinfection efficacy can be significantly reduced when organic load is present as a consequence of improper cleaning prior to their application [13,15,16,17,18,19,20,21]. In practical applications, many times proper cleaning before disinfection is not often employed; thus, understanding the degree to which residual organic load (composed of complex carbohydrates and proteins), can negatively influence the efficacy of commonly used disinfection methods (including, but not limited to, quaternary ammonium compounds, sodium hypochlorite, UV treatment, organic acids, etc.) against both enveloped and non-enveloped viruses is valuable.

Many works on foodborne viral inactivation can often directly evaluate the efficacy of disinfectants on specific foods, which serve as organic loads [22,23,24]. In many cases, food matrices inhibited the effectiveness of disinfection in these tests, so using food could be valuable as a simulated organic load option for laboratory use. Although investigation of the efficacy of disinfectants on viruses on foods is of value, complex components in food matrices utilized can contribute to variability in results. Thus, other research groups utilize simulated organic loads when investigating the efficacy of disinfectants to provide insight into the influence of organic loads in a more controlled, reproducible manner.

Organic load has shown to be a hindrance to bacterial disinfection efficacy in numerous settings [25,26,27,28]. Viruses, especially non-enveloped viruses, are consistently hard to inactivate and behave differently than bacterial pathogens [29]. There is a significant need to understand the impacts that the presence of organic load models has on viral disinfection. The purpose of this review is to survey simulated organic load formulations used in studying foodborne and clinical virus inactivation. This review will present and compare the influence of organic load on viral inactivation in multiple settings. It should be noted that a large body of work on foodborne virus inactivation has been performed and reviewed elsewhere [13,15,16,18,19,20,21,22,23,24,25,26,27,28,29,30,31], thus, this review will focus solely on the influence of simulated organic load on viral inactivation in clinical and food matrices.

## 2. Blood-Based Simulated Organic Load

### 2.1. American Society of Testing and Materials (ASTM) International

In many foodborne virucidal test protocols, ASTM E1052-96 is a widely used standard method for testing the effect of organic load on inactivation of viruses [32]. This method incorporates a serum as organic load [33]. Specifically, calf or other serum in virus suspensions is often used for simulating organic load due to its high concentration of protein and other organic biomolecules [34,35]. In some cases, serum may not be able to be used; for example, fetal bovine serum (FBS) cannot be used with the study of rotaviruses because it is inhibitory toward their replication in vitro [36]. In cases where serum cannot be utilized, alternative protein-rich materials have been investigated to take its place, such as Human Platelet Lysate (HPL) and earthworm Heat-Inactivated Coelomic Fluid (HI-CF) [35].

### 2.2. Serum and Blood Cells

As mentioned above, different concentrations of FBS are frequently used as simulated organic load. Jean et al. [12] utilized 5% FBS in evaluating the inactivation of murine norovirus (MNV-1) and hepatitis A virus (HAV) by pulsed ultraviolet (UV) light (10.5 cm) from the HAV. This work was carried out on 1 cm^2^ stainless steel and polyvinyl chloride (PVC) discs, with treatment for 2 or 3 s in the absence of FBS, resulting in complete inactivation of MNV-1 and HAV (>5-log reduction). In the presence of FBS, HAV was not significantly reduced on either fomite. However, the reduction of MNV-1 on stainless steel and PVC discs in the presence of FBS was 3.6 log and 2.3 log, respectively (Table 2). HAV is a non-enveloped virus, meaning it is more resistant to disinfection than enveloped viruses. Organic load, in this case FBS, significantly interfered with the disinfection of HAV. This study cites that this phenomenon is due primarily to carbohydrates and proteins in FBS. Interfering substances have been highly cited in bacterial applications for their ability to reduce efficacy of disinfectants, which could be similar in viral applications. Similar to the aforementioned research findings, Hudson et al. [9] used 5% FBS as organic load in evaluating the efficacy of ozone gas on feline calicivirus (FCV) on steel, plastic, and glass. The presence of FBS did not significantly affect the reduction of FCV observed with 20–25 parts per million (ppm) ozone gas for 20 min, as >3 log reduction was observed. In addition, the presence of added serum (from a norovirus-positive sample) did not adversely affect the response of FCV to ozone treatment. Although not a foodborne virus, Rabenau et al. [37] interestingly evaluated the potential influence of three different simulated organic load formulations: 0.3% bovine serum albumin (BSA) and 0.3% BSA with 0.3% sheep erythrocyte, against three disinfectants (Mikrobac forte: benzalkonium chloride and lauryl amine; Korsolin FF: benzalkonium chloride, glutaraldehyde, and didecyldimonium chloride; and Dismozon pur: magnesium monoperphthalate) against SARS coronavirus (SARS-CoV) on stainless steel. With all tested preparations, SARS-CoV was inactivated to below the detection limit (reduction factor mostly ≥4). The reduction observed in the presence of these organic loads was the same between these three types of organic load (0.3% BSA, 10% FCS, and 0.3% BSA with 0.3% sheep erythrocyte), even though each exposure time (30 min or 60 min) was different [37]. This high level of reduction removed the ability to compare the influence of the different organic load formulations; however, future work comparing their influence on nonenveloped viruses—which are generally more recalcitrant to inactivation—would be of value.

Another study done by Reissner et al. [41] investigated the overall infectivity of Feline Coronavirus (FCoV) in the presence of yeast-based organic matrices under environmental conditions. The first organic load model used in the study consisted of yeast extract and Bovine Serum Albumin (BSA), and the second consisted of defibrinated sheep blood and BSA. The FCoV was first dried on stainless-steel germ carriers and was subjected to environmental factors such as time and relative humidity. The study found that FCoV was most unstable with the yeast extract/BSA load at 20 °C, with a five log_10_ TCID_50_ reduction achieved after Md = 19 days. With the sheep blood/BSA load at 20 °C, a five log_10_ TCID_50_ reduction was reached after Md = 58 days. At 4 °C, a similar decrease in FCoV concentration was observed with sheep blood/BSA load, where a five log_10_ TCID_50_ reduction was shown after 54 days. This study found that FCoV was most robust in the presence of yeast extract/BSA at 4 °C. It decreased by only three log_10_ levels after 68 days, remaining infectious three times longer than with sheep blood/BSA at 4 °C. This study shows that the presence of organic matter with viruses can aid in their stability, thus allowing them to be infectious on surfaces for longer periods of time.

Ionidis et al. [11] assessed the activity of a novel alcohol-based hand disinfectant in the presence or absence of 10% FBS as an organic load model. This disinfectant contained 69.39% *w*/*w* ethanol, 3.69% *w*/*w* 2-propanol, 3.69% *w*/*w* 2-propanol, 2.0% urea, and 2.0% citric acid. Both nonenveloped [MNV, Adenovirus (AdV), poliovirus, and polyomavirus (PV) SV40] and enveloped [bovine viral diarrhea virus (BVDV), vaccinia virus strain Elstree] viruses were evaluated. The reduction observed for enveloped viruses, and non-enveloped AdV was similar (both displayed a 4.6 log reduction) between purified water and 10% FBS regardless of exposure times. The treatments against PV SV40 and AdV in FBS were not statistically different. The disinfectant inactivated PV and AdV more effectively in 0.3 g/L BSA (the reductions in 1 min were ≥5.75 ± 0.30 and ≥5.38 ± 0.25, respectively) than in FBS (RF in 1 min: 4.31 ± 0.42 and 4.32 ± 0.34, respectively), suggesting that the 10% FBS was more conservative when used as organic load compared to 0.3 g/LBSA.

Another study by Sangsriratanakul et al. [38] investigated alkaline food additive-grade calcium hydroxide (FdCa(OH))_2_ in solution, powder, and suspension forms and evaluated them as virucidal agents against MNV in the presence and absence of fecal organic matter. The results showed that a 0.17% FdCa(OH)_2_ solution could inactivate MNV within 30 s even in the presence of organic materials. In a contaminated surface experiment, MNV with 5% FBS was inoculated on rayon sheets. Results suggest that FdCa(OH)_2_ solution could markedly reduce virus titer within 1 min, even in the presence of organic material. When mouse feces were spiked with MNV and FdCa(OH)_2_ powder as 10% and 20% *w*/*w* were added to the feces, these concentrations could inactivate the virus within 30 min and 15 min, respectively. Whereas, FdCa(OH)_2_ suspension at 2.5% and 5% could inactivate the virus within 30 min and at 1% within 45 min. This ability to maintain efficacy in the presence of organic load is similar to what was observed on Rayon sheets, suggesting that calcium hydroxide may be a promising inactivation agent for use in the presence of organic load, as similar reductions of MNV at 5 and 30 s (approximately 2–2.5 log and 4 log reductions, respectively) with and without 0.1% FBS were observed. However, it should be noted that 0.1% FBS is comparatively in the lower range of what has been used, and an effect may have been observed if a higher concentration was used.

Magulski et al. [19] assessed the activities of three different alcohols (ethanol, 1-propanol, or 2-propanol) against MNV on stainless steel discs. To simulate organic load, virus suspensions were mixed with 0.03% *w*/*v* of BSA or 0.3% BSA plus 0.3% *v*/*v* washed erythrocytes. As a result, there were no significant differences in reduction of MNV with the three alcohols in concentrations of 40% *v*/*v* or 60% *v*/*v* under both organic load conditions. Regardless of organic load levels, 40% *v*/*v* and 60% *v*/*v* 1-propanol and 60% *v*/*v* ethanol exposure for 5 min displayed high efficacy against MNV (approximately six log PFU reduction).

### 2.3. Blood

Alfa & Olson [39] compared organic composition, viscosity, and surface adhesiveness between different types of artificial test soils (ATS) as organic load models with relevant clinical samples from endoscopes. As test soils (organic load models), blood test soil (a blood-based standardized soil that is used for simulated use testing and cleaning validation studies of medical devices), Edinburgh test soil, Edinburgh-M (modified) soil, Miles soil, ATS (formulated as USA Patent #6,447,990 [42]) lyophilized ATS2015, and blood-virus suspension were used. The authors altered the amount of each active component of the organic load samples to ensure the features of the sample in the worst-case scenario were assumed (protein 115 microg/cm^2^, sodium ion 7.4 micromol/cm^2^, hemoglobin 85 microgram/cm^2^, bilirubin 299 nanomol/cm^2^, carbohydrate 29.1 microgram/cm^2^, endotoxin 9852 endotoxin units/cm^2^, and bacteria 7.1 (log_10_ colony-forming units (CFU)/cm^2^), as noted in the study. ATS2015 and Edinburgh-M soils were shown to be appropriate for validating the cleaning of flexible endoscopes as their adhesive characteristics are good, and their viscosity and composition are similar to the worst-case patient secretions to which flexible endoscopes would be exposed. Both of these test soils had protein and hemoglobin levels above the worst-case clinical levels for endoscope channels and exterior surfaces (worst-case levels of protein, carbohydrate, and hemoglobin, and viscosity of clinical material were 219,828 μg/mL, 9296 μg/mL, 9562 μg/mL, and 6 cP, respectively), thereby allowing reliable tracking of the removal of these markers after the cleaning process. The data from this study provides an approach to characterize the adhesiveness of test soils. This data extends knowledge of clinically relevant soiling characteristics so that standardized parameters for test soils used for cleaning validation can be established in the future, potentially for foodborne virus applications. These test soils were not used for the inactivation of viruses. Still, this study displayed that blood soil models could be promising for organic load testing in the study of future foodborne virus inactivation. It is interesting to note that although these blood and serum-based organic load formulations are among the most commonly used for viral inactivation, the degree to which these systems mimic the type of residual organic load likely to be present from foods and in food separation settings has not been the subject of investigation and could be of value to evaluate. That being said, enteric viruses may require organic loading that recreates fecal material in order to be seen as direct models of human conditions, which we will explore in “feces-based simulated organic load” below. Section 1 of Table 2 summarizes the blood-based simulated organic load models that have previously been studied in past published work.

## 3. Feces-Based Simulated Organic Load

In addition to serum- and blood-based simulated organic loads, numerous reports utilize fecal material in different concentrations to serve as organic loads due to its high nitrogen, phosphorus, and potassium content. Mbithi et al., [13] used a 10% fecal suspension negative for foodborne viruses to assess the inactivation of HAV by 20 different disinfectants (specific formulations for each of the 20 disinfectants are referenced in Mbithi et al. [13] on stainless steel). Of these disinfectant formulations, only 2% glutaraldehyde, a quaternary ammonium formulation containing 23% HCI (toilet bowl cleaner), and sodium hypochlorite (>5000 ppm of free chlorine) reduced HAV titer by more than 4 logs (>99.99%) in the presence of the fecal matter for 1 min. All other disinfectants tested were ineffective in reducing HAV titer in the presence of fecal material (most only reduced the HAV by 1 log or less). Though there was no comparison done in this study to non-fecal contaminated surfaces, other published work [43,44,45] suggests that disinfectants and active chemical ingredients similar to those mentioned above are more efficacious against microbial contaminants in the absence of fecal matter, some even prompting the discussion of microbial resistance due to selective pressure of the disinfectant.

Similar to Mbithi et al., Sattar et al. [36] also utilized a 10% fecal suspension to evaluate the efficacy of a disinfectant spray (0.1% o-phenylphenol and 79% ethanol), a domestic bleach (6% sodium hypochlorite diluted to give 800 ppm free chlorine), quaternary ammonium (quat)-based product (7.05% quat diluted 1:128 in tap water), and a phenol-based product (14.7% phenol diluted 1:256 in tap water) against human rotavirus DS-1 on stainless steel and finger pads. The disinfectant spray was applied to the respective surfaces for 3 to 10 min and caused a reduction of the virus by approximately 4 logs (99.99% reduction). The bleach and phenolic acid solutions reduced the virus titer by 97.9% ± 0.4% and 95% ± 5.36%, respectively. Though the presence/absence of the 10% fecal matter was not tested in this study, it is important to note that even in the presence of organic matter, there was still a approximately 4 log reduction of the virus, which could be due to chlorine sequestering the organic material present in the suspension. More work should be done to expand this study to compare the viral reduction with and without the presence of fecal material.

Another study by Sangsriratanakul et al. [38] investigated alkaline food additive-grade calcium hydroxide (FdCa(OH)_2_) in solution, powder, and suspension forms and evaluated them as virucidal agents against MNV in the presence and absence of fecal organic matter. The results showed that 0.17% FdCa(OH)_2_ solution could inactivate MNV within 30 s even in the presence of organic materials (in the form of 5% fetal bovine serum (FBS)). In a contaminated surface experiment, MNV with 5% FBS was inoculated on rayon sheets. Results suggest that FdCa(OH)_2_ solution could markedly reduce virus titer within 1 min, even in the presence of organic material. When mouse feces were spiked with MNV and FdCa(OH)_2_ powder as 10% and 20% *w*/*w* were added to the feces, these concentrations could inactivate the virus within 30 min and 15 min, respectively. Whereas, FdCa(OH)_2_ suspension at 2.5% and 5% could inactivate the virus within 30 min and at 1% within 45 min. This ability to maintain efficacy in the presence of organic load is similar to what was observed with the observation by [38] evaluating calcium hydroxide on Rayon sheets, suggesting that calcium hydroxide may be a promising inactivation agent for use in the presence of organic load.

Alternatively, Park & Sobsey [40] utilized a 10% (*w*/*v*) suspension of GII.4 norovirus-positive stool sample as an organic load when evaluating the efficacy of sodium hypochlorite against FCV, MNV, and bacteriophage MS2 on stainless steel. On fecal soiled surfaces, 5000 ppm of sodium hypochlorite was needed to inactivate FCV, MNV, and MS2 by three logs for 1.9 min, 3.2 min, and 4.5 min of contact time, respectively. Again, the efficacy of disinfectant without organic load was not evaluated, and future work could aim to compare different concentrations of organic load to viral inactivation.

Bosch et al. [10] compared the effect of multiple commercial disinfectants against human rotavirus and HAV dried on polystyrene. The viruses were mixed with 20% sterilized fecal suspension obtained from a healthy male volunteer. Sodium hypochlorite inactivated HAV effectively in PBS (−2.58 ± 1.06 log) but was significantly lower in the presence of the 20% fecal suspension (−1.12 ± 0.53 log). Thus, this test shows that 20% fecal suspension reduced the efficiency of disinfectants significantly.

Hudson et al. [9] investigated the efficacy of ozone gas against norovirus-positive stool samples and FCV on sterile plastic and other surfaces. The norovirus-positive stool was added to FCV in a 1:1 ratio for organic load. Interestingly, viral reduction by ozone gas was not notably different in the presence versus absence of stool (1.54 log and 1.70 logs, respectively) when quantified by RT-qPCR. In the virus plus FBS control, the log reduction with ozone gas was only −1.68, and the virus with the fecal sample log reduction was −1.70, showing that the presence of organic load did not impact the overall efficacy of the ozone gas, similar to what was observed with calcium hydroxide and MNV discussed above, though an effect of organic load has been previously reported in other antimicrobial work with bacteria; thus, more investigation into the influence of organic load on the efficacy of ozone inactivation of noroviruses is needed.

Lloyd-Evans et al. [14] evaluated the efficacy of 27 disinfectant products against human rotavirus in the presence of feces (29.50 g/L as high-added organic load (HOL) and 14.75 g/L as low-added organic load (LOL)) on multiple different fomites (glass, stainless steel, smooth plastic, and rough plastic). Treatments with these different disinfectants were carried out for 1 min. Interestingly, only about a third of the 27 formulations tested resulted in a three or greater log reduction of virus in the presence of fecal material. Generally, the following disinfectant classes or combinations displayed three logs PFU/mL or greater log reduction in this study: glutaraldehyde; quaternary ammonium compounds in combination with (a) alcohols at >40%, or (b) some acids, e.g., HC1, or (c) some bases, e.g., sodium metasilicate; phenols in combination with strong anionic surfactants; chlorine-based disinfectants with free chlorine of >20,000 ppm (iodophors with >10,000 ppm iodine).

In addition to human fecal material, other animal feces have also been used as simulated organic loads when studying viral inactivation. Guan et al. [17] utilized 50% suspension of chicken manure when evaluating the efficacy of bleach, surface decontamination foam (SDF), and Virkon against infectious bursal disease virus (IBDV) on stainless steel. Each disinfectant tested yielded a reduction of Newcastle Disease Virus (NDV) of at least 4.8 log TCID_50_, with the heavy organic preparation at −10 °C for 5 min. However, these reductions were lower than the light organic preparation (preparation of heavy and light organic preparation described in [17]), with which viral titer was reduced by approximately six log TCID_50_. This study suggests that the presence of high organic loads influences the ability of disinfectants to behave effectively against viruses.

Jubinville et al. [46] evaluated the effects of artificial feces, real fecal matter, ASTM tripartite organic load, and fetal bovine serum on the efficacy of thermal inactivation, peracetic acid, and sodium hypochlorite treatment on MNV3. Overall, this work demonstrated that organic matter preserves the integrity of MNV3 after exposure to thermal inactivation, with a viral reduction of approximately 1 log compared to 2.67 in the presence of PBS. However, there was a significantly lower reduction (approximately 2 log PFU/mL) of MNV3 in the presence of peracetic acid and no reduction of MNV3 in the presence of 3% sodium hypochlorite. Based on the results of this work, it was concluded that Feclone™ artificial feces was the most similar to the behavior of real fecal matter. It is possible that there also was an effect on the physical Feclone™ matrix affording protection in addition to the chemical effect of the organic load, though this would require further study. Section 2 of Table 2 summarizes the feces-based simulated organic load models that have previously been studied in past published work.

Another study conducted by Choi et al. [47] investigated the inactivation of Tulane virus, a common norovirus surrogate, in the presence of organic load with aqueous ozone. Major findings from this work indicate that organic load removal is vital for increased viral inactivation prior to ozonation. Removal of organic material in water sources significantly decreases the treatment time of the ozone when decreasing the viral load. This study used Newborn Calf Serum (NCS) as the organic load. The study found that reduction of virus to undetectable levels took up to 36-min of ozonation treatment in the presence of NCS, indicating a significant concern for polluted water contamination.

## 4. Sand and Soil Simulated Organic Load

In addition to biological materials for organic load, sand, and soil have also been used in the study of viral inactivation due to their high nutrient levels essential for cell proliferation. Thompson et al. [20] evaluated the influence of the soil materials Ottawa sand (OS), Tujunga la sand (TLS), and Greenfield sandy loam (GSL) on the inactivation of bacteriophages MS2 and X174 in suspension. Viral persistence in the presence of soil was examined, and ultimately the different soils enhanced the viruses’ ability to persist in the environment. There were, on average, 2.34 log lower concentrations of MS2 in the absence of soil than in the presence of soil. High levels of MS2 inactivation (approximately three log reduction) after 3 h of mixing were observed in control suspensions, but comparatively little loss of virus was seen when soil material was present.

Guan et al., [17] used 5% *w*/*v* sterilized garden soil as organic load for studying viral inactivation on stainless steel. Even in the presence of organic load in the form of soil, SDF and Virkon yielded a five log PFU/mL reduction of IBDV within 15 min at 23 °C and 4 °C. On the other hand, treatment with bleach was required for 2 h to observe a similar reduction as SDF and Virkon. At −20 °C, SDF and Virkon in the presence of garden soil yielded about five logs of PFU/mL reduction of IBDV after 2 h and 24 h. Moreover, bleach was not completely efficacious when inhibiting IBDV at the same temperature in the presence of the organic load. Thus, the garden soil considerably inhibited the efficacy of the three disinfectants, suggesting it may have some utility when studying the inactivation of foodborne viruses, potentially in produce processing settings. In previous studies done on bacterial applications, soil presence has not had as strong an impact on sanitizer performance [48,49]. Thus, this study is significant to our understanding of the behavioral differences between viruses and bacteria in their response to soil-based organic load presence. Section 3 of Table 2 summarizes the sand/soil based simulated organic load models that have previously been studied in past published work.

Another study conducted by Wang et al. [50] studied the disinfection of MNV-1 and HAV by chlorine dioxide and peracetic acid treatments in the presence of soil-rich wash water. This particular study used clay loam and black soil for organic matter matrices, which are common in East Asia where the study was conducted. The study found that with an initial viral titer of 7.58 log_10_ PFU in the simulated black soil wash water, the MNV-1 titer was reduced to 6.34 ± 0.19 log_10_ PFU when treated with 80 ppm peracetic acid (PAA) for 10 min, which is still relatively high and can cause infection. When the PAA concentration was increased to 250 ppm, the MNV-1 titer was reduced to 3.76 ± 0.17 log_10_ PFU for a 10 min treatment. When increasing the PAA concentration to 500 ppm for a 10 min exposure, it resulted in the reduction of the MNV-1 titer to 1.75 ± 0.23 log_10_ PFU (*p* < 0.001). This concentration in a food application setting is not entirely practical. Wash water applications of PAA are from 40–80 ppm [51]. With the initial titer of 7.51 ± 0.13 log_10_ PFU in the clay loam background of the simulated wash water, ideal inactivation of MNV-1 was not achieved at 80 ppm for 1, 5, and 10 min of exposure. The MNV-1 titer was reduced to 2.66 ± 0.15 log_10_ PFU when the PAA concentration was increased to 250 ppm for a 10 min treatment (*p* < 0.001). When the PAA concentration was further increased to 500 ppm after a 10 min treatment, the MNV-1 titer was reduced to 2.03 ± 0.39 log_10_ PFU. This study shows how difficult it can be to remove viral load in organically rich soil matrices. Not only are treatments of any PAA treatment over 80 ppm not recommended in food applications, but they are still relatively ineffective on viruses unless the product is exposed for over 10 min.

## 5. Other Simulated Organic Loads

Gerba et al. [15] investigated the ability of some foodborne viruses to survive on fabrics during laundering. Adenovirus, rotavirus, and HAV were inoculated onto cotton cloth swatches, and these were passed through the wash cycle, the rinse cycle, and a 28-min permanent press drying cycle. To simulate dirty laundry conditions, washing machines contained sterile and virus-inoculated 58 cm^2^ swatches, 3.2 kg of cotton T-shirts and underwear, and a soiled pillowcase. Detergent or detergent with bleach (sodium hypochlorite) was put in the washing machine. In this test, the synthetic organic load (31.2 g serum base, 2.4 g triethandamine, and 3.78 g gelatin) was added to simulate variation (pH, oxidant degradation, solids that cannot be dissolved, turbidity, stain, and soil). When only detergent was used to wash these fabrics, low levels (PFU/mL) of viral reduction were observed (rotavirus: 3.2 log, HAV: 2.99 log, adenovirus: 1.79 log). Detergent with sodium hypochlorite did largely result in effective viral reduction generally with the fabrics (rotavirus: 6.88 log, HAV: 6.98 log, adenovirus: 4.38 log). However, given the effects of bleach on many fabrics, the practical utilization of it is limited with this application.

Another study done by Qi et al. [52] discusses the affinity of coronaviruses to bind with contact surfaces in the presence of simulated body fluids (saliva and urine). This study is especially pertinent to food service applications within the food industry, as many sources of contamination come from contaminated employees handling high touch surfaces. The study found that since saliva contains mucin, it was more easily removed from a surface containing coronavirus than urine was, but both can attach themselves to surfaces more readily if left for longer periods of time. From these findings, it can be concluded that prompt cleaning and sanitation practices in the food industry can significantly minimize viral transmission. The study also found that higher adhesion occurred on stainless steel surfaces. This is particularly important for the food industry, as 30% of all stainless steel products made today are used in the food industry [53].

Another study done by Vinnerås et al. [54] discusses the effects of modified human urine on the inactivation of viruses, specifically MS_2_ bacteriophage, a norovirus surrogate. In this study, urine was diluted in water at 1:0, 1:1, and 1:3 ratios, respectively, and was stored at either 4, 14, 24, and 34 °C. The concentration of ammonia in the urine was also adjusted to 40 mM NH_3_ in all samples. In the presence of urine at temperatures below 20 °C, bacteriophage reduction slowed dramatically. Therefore, a study found that urine stored at temperatures below 20 °C carries a high risk of containing viable viruses, and suggests that ammonia content in urine can significantly impact the level of viable virus in urine samples.

Zuo et al. [55] investigated the inactivation of airborne MS_2_ bacteriophage in the presence of simulated human saliva (recipe derived from Woo et al. [56]). The study found that the relative recovery of infectious virus was comparable to the recovery of infectious virus in TSB, a commonly used growth media. It was found that mucin content in artificial saliva contributed to the overall preservation of the virus, even through the process of aerosolization. Mucin’s cross-linking attributes form a layer over the virus during aerosolization, this protecting it from external inactivators.

## 6. Conclusions

This review suggests that many simulated organic load formulations can reduce disinfectants’ efficacy against viruses, both non-enveloped and enveloped. Based on the findings in this review, blood, particularly serum or feces, are among the most commonly used and efficacious forms of simulated organic load in many tests. Organic loads were often chosen for specific experiments due to the nature of the application, contamination level, and the conditions of everyday life. Future work should address the significant need for organic load formulations reflecting specific food types, and see if results or the effects differ from those observed with the simulated organic load formulations discussed here. If so, specific formulations will be needed to better evaluate organic load in food applications of disinfectant. Similarly, vomitus is important for transmission of norovirus, and development and investigation of a simulated vomitus model on nonenveloped virus inactivation would be of value. Finally, although stool and urine simulated matrices have been discussed, the development and consideration of simulated organic loads mimicking wastewater would be especially valuable in understanding and evaluating novel wastewater treatment strategies. Consideration of using the aforementioned simulated organic load formulations’ effect on the downstream quantification of viruses should be further examined. Another major consideration in comparing the inactivation of these viruses in addition to and in the context of the presence of organic load is whether viral inactivation is evaluated on surfaces like stainless steel and PVC or if it is in suspension. In summary, organic load is an important consideration when evaluating disinfectant behavior against viruses, and choosing the correct organic load for a specific application should be considered when evaluating the efficacy of an inactivation agent against foodborne viruses. Since many studies currently available today do not discuss the efficacy of disinfectants with and without the presence of organic load, future work should aim to facilitate the comparison of different concentrations of organic load in relation to viral inactivation.

## Figures and Tables

**Table 1:** Comparison of the influence of organic load on viral inactivation of various inactivation agents under different conditions. Brackets around reduction standard deviation indicate statistical significance.

Virus Tested	Treatment	Condition (Time, pH)	Reduction (log_10_)		Reference
			No Organic Load	Organic Load	
Feline calicivirus (FCV)	Ozone gas	20–25 ppm, 20 min	3.17	3.03 (FBS)	[9]
				3.01 (Stool)	
Hepatitis A virus (HAV)	Ethanol	70% 10 min, pH 7.43	1.36 (±0.25)	1.33 (±0.24)	[10]
	Chlorhexidine di-gluconate	0.05% 10 min, pH 7.75	0.95 (±0.47)	0.52 (±0.40)	
	Sodium hypochlorite	0.125% 10 min, pH 9.56	2.58 (±1.06)	1.12 (±0.53)	
	Phenol + Sodium hypochlorite	1.41% + 0.24% pH 8.21	2.09 (±0.77)	1.66 (±0.78)	
	Diethylenetriamine	0.0192% pH 11.86	2.61 (±0.55)	1.67 (±0.68)	
	Sodium Chlorine	30% pH 1.85	3.71 (±1.31)	2.91 (±0.18)	
Human rotavirus (HRV)	Ethanol	70% 10 min, pH 7.43	2.34 (±0.65)	0.91 (±0.56)	
	Chlorhexidine di-gluconate	0.05% 10 min, pH 7.75	0.82 (±0.49)	0.45 (±0.58)	
	Sodium hypochlorite	0.125% 10 min, pH 9.56	2.76 (±1.14)	1.62 (±0.94)	
	Phenol + Sodium hypochlorite	1.41% + 0.24% pH 8.21	0.87 (±0.83)	0.19 (±0.12)	
	Diethylenetriamine	0.0192% pH 11.86	1.93 (±0.65)	1.32 (±0.63)	
	Sodium Chlorine	30% pH 1.85	2.96 (±0.89)	1.41 (±0.64)	
Bacteroides fragilis bacteriophages (BFB)	Ethanol	70% 10 min, pH 7.43	0.19 (±0.12)	0.62 (±0.64)	
	Chlorhexidine di-gluconate	0.05% 10 min, pH 7.75	0.22 (±0.12)	0.71 (±0.08)	
	Sodium hypochlorite	0.125% 10 min, pH 9.56	1.25 (±0.27)	0.67 (±0.05)	
	Phenol + Sodium hypochlorite	1.41% + 0.24% pH 8.21	0.54 (±0.21)	0.22 (±0.03)	
	Diethylenetriamine	0.0192% pH 11.86	2.29 (±1.51)	0.33 (±0.06)	
	Sodium Chlorine	30% pH 1.85	5.81 (±0.15)	3.19 (±0.84)	
Poliovirus	Hand disinfectant	69.39% *w*/*w* ethanol, 3.69% *w*/*w* 2-propanol, 2.0% urea, and 2.0% citric acid, 60 s	≥6.13 ± (0.35)	4.57 ± (0.37)	[11]
Adenovirus type 5 (ADV)	Hand disinfectant	69.39% *w*/*w* ethanol, 3.69% *w*/*w* 2-propanol, 2.0% urea, and 2.0% citric acid, 90 s	≥5.13 ± (0.33)	4.94 ± (0.40)	
Polyomavirus SV40 (SV40)	Hand disinfectant	69.39% *w*/*w* ethanol, 3.69% *w*/*w* 2-propanol, 2.0% urea, and 2.0% citric acid, 30 s	≥5.44 ± (0.27)	3.75 ± (0.55)	
Murine norovirus (MNV)	Pulsed Ultraviolet (UV)	2 s at 10.5 cm from the UV source (0.060 W s/cm^2^) on stainless steel	5.0	3.6 (±1.2)	[12]
		2 s at 10.5 cm from the UV source (0.060 W s/cm^2^) on polyvinyl chloride (PVC)	5.0	2.3 (±1.2)	

**Table 2:** Previously studied organic load model types with general concentrations.

Organic Load		Concentration	Reference
**Blood**	Bovine serum albumin (BSA)	0.3%, 0.03%, or 0.3 g/L BSA	[19]
			[37]
			[11]
	Fetal calf serum (FCS)	10% FCS	[37]
	BSA and sheep erythrocyte	0.3% BSA with 0.3% sheep erythrocyte	[37]
			[11]
			[19]
	Fetal bovine serum (FBS)	5% FBS	[9]
			[12]
			[38]
	Blood test soil	Heparinized sheep blood with 1 mg protamine sulphate per 1 mL of either whole or 10% heparinized sheep blood	[39]
	Edinburgh test soil	100 mL egg yolk, 10 mL defibrinated sheep blood, and 2 g hog mucin	
	Edinburgh-M (modified) soil	100 mL egg yolk, 100 mL defibrinated sheep blood, and 2 g hog mucin	
	Miles soil	1 mL sheep blood, 6 g dry milk powder, 10 mL FCS, and 1 mL saline (0.9% NaCl)	
	Artificial test soil (ATS)	Base medium (up to 20% *v*/*v* serum; up to 10% *v*/*v* blood and up to 2,000,000 EU/mL endotoxin), about 1000 nanomoles/mL of the bilirubin and up to 10,000 µg/mL of the mucin or carbohydrate	
	The lyophilized ATS2015 version	A physiological salt (mucin, insoluble cellulose fiber, and reconstituted dried egg yolk; the subsequent addition of 20% sterile sheep blood)	
**Fecal**	Fecal (stool) suspension	10% suspension of feces in normal saline	[13]
			[36]
		A 10% (*w*/*v*) stool suspension consisted of 100 mg/mL solid human stool having MS2, FCV, and MNV	[40]
		20% fecal suspension in PBS	[10]
	Feces with Human rotavirus (HRV)	HRV was suspended in four different fecal samples from laboratory-confirmed cases of rotaviral diarrhea	[14]
	NV positive stool	N/A	[9]
	Chicken manure	5% *w*/*v* chicken manure	[17]
**Sand/Soil**	Ottawa sand (OS)	No clay fraction, and an organic carbon content of 0 mg	[20]
	Loamy sand (TLS)	6.7 mg of organic carbon, 4.5% clay	
	Greenfield sandy loam (GSL)	9.21 mg of organic matter, 9.5% clay	
	Heavy organic load	5% *w*/*v* garden soil	[17]
**Others**	Peptide and protein	A peptide and protein mixture	[17]
	Synthetic organic load	31.2 g sebum base, 2.4 g triethandamine, and 3.78 g gelatin	[15]

## References

[1] Barker J., Stevens D., Bloomfield S. (2001). Spread and prevention of some common viral infections in community facilities and domestic homes. J. Appl. Microbiol..

[2] Jones M.K., Watanabe M., Zhu S., Graves C.L., Keyes L.R., Grau K.R., Gonzalez-Hernandez M.B., Iovine N.M., Wobus C.E., Vinjé J. (2014). Enteric bacteria promote human and mouse norovirus infection of B cells. Science.

[3] Lanata C.F., Fischer-Walker C.L., Olascoaga A.C., Torres C.X., Aryee M.J., Black R.E. (2013). Global Causes of Diarrheal Disease Mortality in Children <5 Years of Age: A Systematic Review. PLoS ONE.

[4] Boone S.A., Gerba C.P. (2007). Significance of Fomites in the Spread of Respiratory and Enteric Viral Disease. Appl. Environ. Microbiol..

[5] Havelaar A.H., Kirk M.D., Torgerson P.R., Gibb H.J., Hald T., Lake R.J., Praet N., Bellinger D.C., de Silva N.R., Gargouri N. (2015). World Health Organization Global Estimates and Regional Comparisons of the Burden of Foodborne Disease in 2010. PLoS Med..

[6] Glass R.I., Parashar U.D., Estes M.K. (2009). Norovirus Gastroenteritis. N. Engl. J. Med..

[7] Lopman B., Gastañaduy P., Park G.W., Hall A.J., Parashar U.D., Vinjé J. (2012). Environmental transmission of norovirus gastroenteritis. Curr. Opin. Virol..

[8] Lin Q., Lim J.Y.C., Xue K., Yew P.Y.M., Owh C., Chee P.L., Loh X.J. (2020). Sanitizing agents for virus inactivation and disinfection. View.

[9] Hudson J., Sharma M., Petric M. (2007). Inactivation of Norovirus by ozone gas in conditions relevant to healthcare. J. Hosp. Infect..

[10] Bosch A., Abad F.X., Pinto R.M. (1997). Disinfection of human enteric viruses on fomites. FEMS Microbiol Lett..

[11] Ionidis G., Hübscher J., Jack T., Becker B., Bischoff B., Todt D., Hodasa V., Brill F.H.H., Steinmann E., Steinmann J. (2016). Development and virucidal activity of a novel alcohol-based hand disinfectant supplemented with urea and citric acid. BMC Infect. Dis..

[12] Jean J., Morales-Rayas R., Anoman M.-N., Lamhoujeb S. (2010). Inactivation of hepatitis A virus and norovirus surrogate in suspension and on food-contact surfaces using pulsed UV light (pulsed light inactivation of food-borne viruses). Food Microbiol..

[13] Mbithi J.N., Springthorpe V.S., Sattar S.A. (1990). Chemical Disinfection of Hepatitis A Virus Environmental Surfaces. Appl. Environ. Microbiol..

[14] Lloyd-Evans N., Springthorpe V.S., Sattar S.A. (1986). Chemical disinfection of human rotavirus-contaminated inanimate surfaces. Epidemiology Infect..

[15] Gerba C.P., Kennedy D. (2007). Enteric Virus Survival during Household Laundering and Impact of Disinfection with Sodium Hypochlorite. Appl. Environ. Microbiol..

[16] Park G.W., Boston D.M., Kase J.A., Sampson M.N., Sobsey M.D. (2007). Evaluation of Liquid- and Fog-Based Application of Sterilox Hypochlorous Acid Solution for Surface Inactivation of Human Norovirus. Appl. Environ. Microbiol..

[17] Guan J., Chan M., Brooks B.W., Rohonczy L. (2014). Inactivation of Infectious Bursal Disease and Newcastle Disease Viruses at Temperatures Below 0 C Using Chemical Disinfectants. Avian Dis..

[18] Kamarasu P., Hsu H.-Y., Moore M.D. (2018). Research Progress in Viral Inactivation Utilizing Human Norovirus Surrogates. Front. Sustain. Food Syst..

[19] Magulski T., Paulmann D., Bischoff B., Becker B., Steinmann E., Steinmann J., Goroncy-Bermes P., Steinmann J. (2009). Inactivation of murine norovirus by chemical biocides on stainless steel. BMC Infect. Dis..

[20] Thompson S.S., Flury M., Yates M.V., Jury W.A. (1998). Role of the air-water-solid interrace in bacteriophage sorption experiments. Appl. Environ. Microbiol..

[21] Weber D.J., Barbee S.L., Sobsey M.D., Rutala W.A. (1999). The effect of blood on the antiviral activity of sodium hypochlorite, a phenolic, and a quaternary ammonium compound. Infect Control Hosp Epidemiol..

[22] Hirneisen K., Markland S., Kniel K. (2011). Ozone Inactivation of Norovirus Surrogates on Fresh Produce. J. Food Prot..

[23] Huang Y., Ye M., Cao X., Chen H. (2017). Pulsed light inactivation of murine norovirus, Tulane virus, Escherichia coli O157:H7 and Salmonella in suspension and on berry surfaces. Food Microbiol..

[24] Li X., Ye M., Neetoo H., Golovan S., Chen H. (2013). Pressure inactivation of Tulane virus, a candidate surrogate for human norovirus and its potential application in food industry. Int. J. Food Microbiol..

[25] Ayyildiz O., Ileri B., Sanik S. (2009). Impacts of water organic load on chlorine dioxide disinfection efficacy. J. Hazard. Mater..

[26] Codling C.E., Maillard J.-Y., Russell A.D. (2003). Performance of Contact Lens Disinfecting Solutions Against Pseudomonas aeruginosa In the Presence of Organic Load. Eye Contact Lens Sci. Clin. Pr..

[27] Williams G.J., Denyer S.P., Hosein I.K., Hill D.W., Maillard J.-Y. (2009). Limitations of the Efficacy of Surface Disinfection in the Healthcare Setting. Infect. Control Hosp. Epidemiol..

[28] Yokoyama T., Miyazaki S., Akagi H., Ikawa S., Kitano K. (2021). Kinetics of bacterial inactivation by peroxyni-tric acid in the presence of organic contaminants. Appl Env. Microbiol. Am. Soc. Microbiol..

[29] Australia H. What Is the Difference between Bacterial and Viral Infections?. Healthdirect Australia 2023..

[30] Hirneisen K.A., Kniel K.E. (2013). Comparing Human Norovirus Surrogates: Murine Norovirus and Tulane Virus. J. Food Prot..

[31] Li J., Predmore A., Divers E., Lou F. (2012). New Interventions Against Human Norovirus: Progress, Opportunities, and Challenges. Annu. Rev. Food Sci. Technol..

[32] Bouchard S., Paniconi T., Jubinville É., Goulet-Beaulieu V., Goetz C., Marchand P., Jean J. (2023). Inactivation of foodborne viruses by novel organic peroxyacid-based disinfectants. Front. Microbiol..

[33] Standard Test Method for Sandwich Corrosion Test. https://standards.iteh.ai/catalog/standards/astm/3a3596a4-3735-4c79-9758-9c809f5bc7c6/astm-f1110-02.

[34] Fisher H.W., Puck T.T., Sato G. (1958). Molecular Growth Requirements of Single Mammalian Cells: The Action of Fetuin in Promoting Cell Attachment to Glass. Proc. Natl. Acad. Sci. USA.

[35] Chelladurai K.S., Rajagopalan K., Yesudhason B.V., Venkatachalam S., Mohan M., Vasantha N.C., Christyraj J.R.S.S. (2021). Alternative to FBS in animal cell culture—An overview and future perspective. Heliyon.

[36] Sattar S.A., Jacobsen H., Rahman H., Cusack T.M., Rubino J.R. (1994). Interruption of Rotavirus Spread through Chemical Disinfection. Infect. Control Hosp. Epidemiol..

[37] Rabenau H., Kampf G., Cinatl J., Doerr H. (2005). Efficacy of various disinfectants against SARS coronavirus. J. Hosp. Infect..

[38] Sangsriratanakul N., Toyofuku C., Suzuki M., Komura M., Yamada M., Alam M.S., Ruenphet S., Shoham D., Sakai K., Takehara K. (2018). Virucidal efficacy of food additive grade calcium hydroxide against surrogate of hu-man norovirus. J. Virol. Methods.

[39] Alfa M., Olson N. (2016). Physical and composition characteristics of clinical secretions compared with test soils used for validation of flexible endoscope cleaning. J. Hosp. Infect..

[40] Park G.W., Sobsey M.D. (2011). Simultaneous Comparison of Murine Norovirus, Feline Calicivirus, Coliphage MS2, and GII.4 Norovirus to Evaluate the Efficacy of Sodium Hypochlorite Against Human Norovirus on a Fecally Soiled Stainless Steel Surface. Foodborne Pathog. Dis..

[41] Reissner J., Siller P., Bartel A., Roesler U., Friese A. (2023). Stability of Feline Coronavirus in aerosols and dried in organic matrices on surfaces at various environmental conditions. Sci. Rep..

[42] Anonymous 1999 Artificial Test Soil. https://patents.google.com/patent/US6447990B1/en.

[43] Bardi M.J., Oliaee M.A. (2021). Impacts of different operational temperatures and organic loads in anaerobic co-digestion of food waste and sewage sludge on the fate of SARS-CoV-2. Process Saf. Environ. Prot..

[44] Mcdonnell G., Russell A.D. (1999). Antiseptics and Disinfectants: Activity, Action, and Resistance. Clin Microbiol Rev..

[45] de Souza C., Mota H.F., Faria Y.V., Cabral F.d.O., de Oliveira D.R., Sant’anna L.d.O., Nagao P.E., Santos C.d.S., Moreira L.O., Mattos-Guaraldi A.L. (2020). Resistance to Antiseptics and Disinfectants of Planktonic and Biofilm-Associated Forms of *Corynebacterium striatum*. Microb. Drug Resist..

[46] Jubinville E., Girard M., Trudel-Ferland M., Fliss I., Jean J. (2021). Inactivation of Murine Norovirus Suspended in Organic Matter Simulating Actual Conditions of Viral Contamination. Food Environ. Virol..

[47] Choi J.M., D’Souza D.H. (2023). Inactivation of Tulane virus and feline calicivirus by aqueous ozone. J. Food Sci..

[48] Wang Y., Jin Y., Han P., Hao J., Pan H., Liu J. (2021). Impact of Soil Disinfestation on Fungal and Bacterial Communities in Soil With Cucumber Cultivation. Front. Microbiol..

[49] Anonymous FS14/FS077: Basic Elements of Equipment Cleaning and Sanitizing in Food Processing and Handling Operations. https://edis.ifas.ufl.edu/publication/FS077.

[50] Wang Z., Yeo D., Kwon H., Zhang Y., Yoon D., Jung S., Hossain I., Jeong M.-I., Choi C. (2024). Disinfection efficiency of chlorine dioxide and peracetic acid against MNV-1 and HAV in simulated soil-rich wash water. Food Res. Int..

[51] Anonymous Vegetable: Produce Wash Water Sanitizers: Chlorine and PAA|Center for Agriculture, Food, and the Environment at UMass Amherst. https://ag.umass.edu/vegetable/fact-sheets/produce-wash-water-sanitizers-chlorine-paa.

[52] Qi Y., Guan X., Shen Y., Liu X. (2024). Probing the Affinity of Coronavirus with Contact Surfaces in Simulated Body Fluids. Environ. Health.

[53] Anonymous How Is Stainless Steel Used in the Food Service Industry?. https://www.meadmetals.com/blog/stainless-steel-food-industry.

[54] Vinnerås B., Nordin A., Niwagaba C., Nyberg K. (2008). Inactivation of bacteria and viruses in human urine depending on temperature and dilution rate. Water Res..

[55] Zuo Z., Kuehn T.H., Bekele A.Z., Mor S.K., Verma H., Goyal S.M., Raynor P.C., Pui D.Y.H. (2014). Survival of Airborne MS2 Bacteriophage Generated from Human Saliva, Artificial Saliva, and Cell Culture Medium. Appl. Environ. Microbiol..

[56] Woo M.-H., Hsu Y.-M., Wu C.-Y., Heimbuch B., Wander J. (2010). Method for contamination of filtering facepiece respirators by deposition of MS2 viral aerosols. J. Aerosol Sci..

